# Gait improvements by assisting hip movements with the robot in children with cerebral palsy: a pilot randomized controlled trial

**DOI:** 10.1186/s12984-020-00712-3

**Published:** 2020-07-03

**Authors:** Shihomi Kawasaki, Koji Ohata, Takeshi Yoshida, Atsushi Yokoyama, Shigehito Yamada

**Affiliations:** 1 Department of Physical Therapy, Human Health Sciences, Kyoto University Graduate School of Medicine, Kyoto, Japan; 2 Department of Pediatrics, Kyoto University Graduate School of Medicine, Kyoto, Japan; 3 Congenital Anomaly Research Center, Kyoto University Graduate School of Medicine, Kyoto, Japan

**Keywords:** Gait, Cerebral palsy, Rehabilitation robot, Exoskeleton, Kinematics, Kinetics

## Abstract

**Background:**

Recently, rehabilitation robots are expected to improve the gait of cerebral palsy (CP) children. However, only few previous studies have reported the kinematic and kinetic changes by using wearable exoskeleton robots. The aim of this study was to investigate the change in gait parameters in CP children by training with the wearable robot-assisted gait training.

**Methods:**

10 spastic CP children with Gross Motor Function Classification Scale levels I-III completed a sham-controlled crossover randomized trial. Robot-assisted gait training (RAGT) and non-assisted gait training (NAGT) were performed on the treadmill with the Honda Walking Assist (HWA) in two different days. To examine the carry-over effect from treadmill walking to overground walking, participants also performed 5.5 m overground-walks without the HWA before and after treadmill training (pre- and post-trial). During treadmill walking, peak of both hip and knee angles were measured. Also, we calculated the limb symmetry of hip range of motion. In addition, gait speed and ground reaction force were measured in overground trials.

**Results:**

The maximum hip angle on the limb with fewer hip movements, which was defined as the affected limb, showed a significant interaction between ASSIST (RAGT and NAGT) and TIME (pre- and post-trial) (*p* < 0.05). Limb symmetry significantly improved after RAGT (p < 0.05), but not in NAGT. Furthermore, the affected limb showed a significant increase in the positive peak of the anterior-posterior ground reaction force during 70–100% of the gait cycle (*p* < 0.05). However, there was no change in gait speed.

**Conclusion:**

By assisting the both hip movements with the HWA, maximum hip flexion and extension angle of the affected limb improved. Also, limb symmetry and propulsion force of the affected limb improved. Our results suggest that assisting both hip movements with the HWA might be an effective method for improving gait in CP children.

**Trial registration:**

UMIN-CTR, UMIN000030667. Registered 3 January 2018, https://upload.umin.ac.jp/cgi-open-bin/ctr_e/ctr_view.cgi?recptno=R000033737

## Background

Cerebral palsy (CP) is a motor dysfunction due to nonprogressive neurological brain disorder in early development [1]. Many children with spastic CP have gait difficulties that inhibit them from walking in daily life. For example, most children with CP walk with abnormal gait patterns such as equinus or crouch gait [2]. These abnormal gait patterns may lead to secondary impairments and low quality of life [3]. Also, a previous survey for adults with CP showed that almost 38% of this population had lost their gait ability before reaching adulthood [4]. Therefore, developing strategies for maintaining their gait function for a long period is crucial for the rehabilitation of children with CP.

Recently, some studies have investigated the effects of rehabilitation robots for improving the gait function in CP. The previous review about robot-assisted gait training (RAGT) for children with CP have reported that RAGT was effective for increasing gait speed, gait endurance, and gross motor function [5]. Also, most previous studies have reported the effect of RAGT on CP children while using the stationary type of robots such as Lokomat, but little is known about the effect of wearable exoskeleton robots. Furthermore, there are only few reports that have investigated the changes in gait kinematics and kinetics induced by wearable exoskeleton robots. Lerner, for example, reported that the lower-extremity exoskeleton they have developed were able to change the kinematics of the knee joint by assisting the knee movements [6]. This study, however, did not investigate changes in other joint movements or kinetics. Further development and investigation of an effective rehabilitation robot to change the gait kinematics and kinetics in children with CP are necessary.

Honda Walking Assist (HWA) [7–10] is a mobile exoskeleton type of robot that assists hip flexion and extension of both limbs during gait, developed by Honda R&D Co., Ltd. The HWA assists only a single joint and does not limit the degree of freedom on other joints, which would be effective for sufficient locomotor learning. A previous study reported that the HWA was effective for reducing energy expenditure in healthy young adults [7]. Additionally, the HWA was able to increase the symmetry of hip movements [8] and paretic step length [9] in stroke individuals. Furthermore, chronic stroke individuals were able to improve walking endurance and increase steps in daily life activity in comparison to functional gait training [10]. As most children with CP has neurological problems on both legs, we hypothesized that assisting both hip movements with the HWA would be an effective way for children to learn the symmetrical gait pattern. This study aimed to compare the differences in gait speed, gait kinematic and kinetic parameters in children with CP by training with and without the HWA.

Also, many studies have focused on the wearing effect of the rehabilitation robots. Some studies have reported that changes induced by the robots remained even after taking them off. Remain of the learning effect is very important since most patients use rehabilitation robots only in clinical settings. Thus, we have evaluated not only the immediate effects but also the learning effect of the HWA in this study.

## Methods

### Participants

In total, 11 children aged 5 to 16 years and diagnosed with spastic cerebral palsy were recruited in this study. The doctor enrolled and assigned the children to participate in the study. All participants were in the Gross Motor Function Classification System (GMFCS) levels I to III, able to walk for 500 m with or without any help, able to understand the method of the trial, and fit the size of the HWA. Children who had pain due to an orthopedic diseases, cardiopulmonary disease, or absence of understanding the trial method were excluded. Written informed consent was obtained from the participants and their parents for the research and the publication of its results. All procedures were approved by the Ethics Committee of Kyoto University Graduate School and Faculty of Medicine (C1313). The trial registration number is UMIN000030667.

### Honda walking assist

The HWA consists of a lumbar part with batteries, two actuators, and two thigh frames. The device weighed approximately 2.7 kg. The actuators are on the lateral side of each hip joint and produce hip flexion and extension assistive torques, with a maximum assistive torque of 4 Nm. The HWA detects the gait cycle by the potentiometers (set beside the actuators) and produces flexion and extension torques on swing and stance phases, respectively. Because the HWA was primarily designed for adults, the lumbar part and thigh frames used in this study were modified to fit the children’s body.

### Procedures

This study was a sham-controlled crossover randomized trial, and was carried out on two different days at intervals of a few weeks. Participants were randomly enrolled into two groups: Group 1 received robot-assisted gait training (RAGT) on the first day and non-assisted gait training (NAGT) on the second day, and Group 2 received NAGT on the first day and RAGT on the second day. Simple randomization was done by generating a list of two computerized random numbers for each participant. We repeated the randomization until the number of the participants in both groups became equal.

For each day, participants first performed two 5.5 m overground-walks (pre-trial) without wearing the HWA and a single 30 s treadmill walk wearing the HWA with no assistive torque. Thereafter, the participants randomly performed 30s walking trials (10 times) either with (RAGT) or without (NAGT) assistive torque, according to the group they were enrolled in. Before starting the RAGT, the assistive torque was adjusted to symmetrize the limb movements. After RAGT or NAGT trial, five 30 s treadmill walking trials with no assistive torque of the HWA were performed to wash out the immediate effects which will soon disappear. Then, two 5.5 m overground-walks (post-trial) without the HWA were performed. During overground-walks, gait speed was measured as a primary outcome measure, and kinematic and kinetic parameters were measured as secondary outcome measures. Clinical assessments were done on the first day. All the data was collected in Kyoto University. For the treadmill settings, the inclination degree of the treadmill was 0%. Also, the treadmill speed was determined by the walking speed in pre-trial for each participant. However, there was a maximum limit for the treadmill speed due to the mechanical problem of the HWA.

### Clinical assessments

Modified Ashworth Scale (MAS) and range of motion (ROM) of hip flexion, hip extension, knee flexion, knee extension, dorsiflexion, and plantar flexion on both limbs were measured as indicators of spasticity and joint flexibility. To investigate the muscle strength, maximum voluntary isometric contraction (MVC) of hip flexor, knee flexor, knee extensor, ankle dorsiflexor, and plantar flexor muscles on both limbs were measured together. The measured torque was multiplied by the moment arm and normalized by the weight of each participant (Nm/kg). Dimensions D and E of Gross Motor Function Measure-88 (GMFM-88) were measured to assess standing and walking ability. Pediatric Evaluation of Disability Inventory (PEDI) was measured to assess the capability and functional performance of mobility in daily life.

### Kinematic and kinetic parameters during gait

Both lower limb movements during overground walking trials were measured using Xsens MVN BIOMECH Awinda inertial sensors (Xsens Technologies, Enschede, the Netherlands) with full body configuration. The sampling rate was 60 Hz. The data was analyzed using MVN Studio Biomech. Each maximum and minimum peak angles were calculated from the averaged data at least five strides. The peaks were defined as maximum flexion (MHF, MKF), maximum extension (MHE, MKE), respectively. Also, the range of motion were defined as H-ROM and K-ROM. Furthermore, we separated the limbs into two groups according to the H-ROM of pre-trial. The limb with smaller hip movements was defined as the affected limb, and the other limb with larger hip movements was defined as the unaffected limb. The limb symmetry (LS) was calculated by dividing the H-ROM of the affected limb by that of the unaffected limb.

The ground reaction force of anterior-posterior torque and vertical torque were measured and recorded for overground trials by the AMTI force plates (AMTI Japan, Japan) at a sampling rate of 1000 Hz. Anterior-posterior torque was defined as Fy, and vertical torque was defined as Fz in this study. Two force plates were placed on either side of the walkway centerline. The data was imported into Matlab 2017b (MathWorks Inc., USA). To remove the electrical noise and detect the correct peak, data of ground reaction force was smoothed by Savitzky-Golay smoothing filter [11, 12]. This filter has an advantage of removing the noise data without distorting the signal tendency. The filtered data was normalized with the stance phase time. For Fy, three to four averaged data of negative peak in the 0–30% gait cycle and positive peak in the 70–100% gait cycle were defined as Fy1 and Fy2, respectively. Fy1 represents the braking force and Fy2 represents the propulsion force. For Fz, a positive peak in 0–30% and 70–100% were also defined as Fz1 and Fz2. Fz1 and Fz2 represent the amount of load on each limb for each double support time.

### Statistical analysis

IBM SPSS version 23 (IBM SPSS INC., USA) was used for all statistical analyses. As the sample size of each group was very limited, we did not perform statistical analysis for comparing the demographics in two groups.

To evaluate the effect of the robot, kinematic (MHF, MHE, MKF, MKE, H-ROM, K-ROM, LS) and kinetic (Fy1, Fy2, Fz1, and Fz2) parameters, and gait speed in overground trials were statistically analyzed. Furthermore, three-way repeated-measures ANOVA was used to compare the MHF, MKF, MHE, MKE, H-ROM, K-ROM of overground trials by ASSIST (RAGT and NAGT), SIDE (affected and unaffected), TIME (pre-trial and post-trial). LS was compared using the Wilcoxon signed-rank test. Also, three-way repeated-measures ANOVA was used to compare Fy1, Fy2, Fz1, and Fz2 by ASSIST (RAGT and NAGT), SIDE (affected and unaffected) and TIME (pre-trial and post-trial) for the ground reaction force analysis. Multiple comparisons were performed after each three-way repeated-measures ANOVA. Gait speed of pre- and post-trial were also compared using the paired t-test. Also, to investigate the effect of the treadmill speed setting on each participant, we used the Pearson correlation coefficient to determine the relationship between the average gait speed in pre-trial, and the difference in treadmill speed and average gait speed during pre-trial.

## Results

**Fig. 1:**
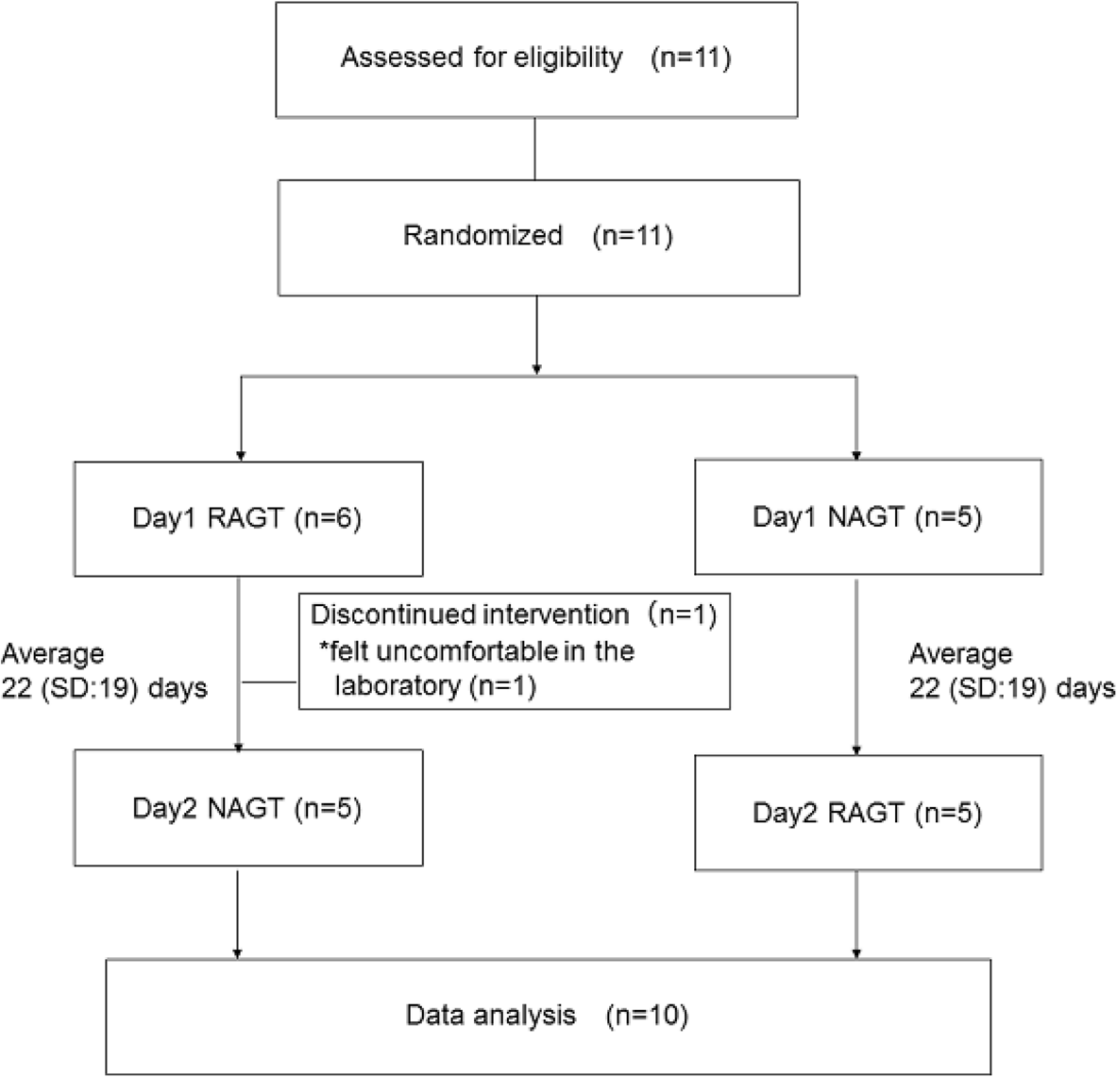
Flow diagram of the subjects in this study

10 children finished all the trials, and 1 child was not able to complete the measurements because the child felt uncomfortable in the laboratory (Fig. 1). The mean duration between the two measurement days is 22 (SD: 19). Also, there were no important harms or unintended effects while measurements in both groups. The characteristics of the participants are shown in Table 1. For H-ROM in the pre-trial, 6 participants had a smaller range of motion in the right limb and 4 in the left limb.

**Table 1:** Participant demographics

	All *n* = 10	Group1 *n* = 5	Group2 *n* = 5
Age (SD)	11.1 (2.3)	10.9 (3.0)	11.4 (1.7)
Gender (male / female)	4 / 6	2 / 3	2 / 3
Height (SD),cm	135.2 (15.1)	130.4 (15.6)	140.1 (14.5)
Weight (SD),kg	31.8 (8.7)	31.1 (8.9)	32.4 (9.4)
CP distribution, n			
Diplegia	7	3	4
Hemiplegia	3	2	1
GMFCS level (I / II / III)	2 / 5 / 3	2 / 0 / 3	0 / 5 / 0
GMFM Dimensions D & E (SD)	64.2 (27.7)	53.2 (27.6)	75.2 (25.7)
PEDI (SD)	45.2 (8.2)	46.2 (10.0)	44.2 (6.9)
Affected limb (R / L)	6 / 4	4 / 1	2 / 3

Group1 performed robot-assisted gait training on the first day, and non-assisted gait training on the second day. Group2 performed non-assisted gait training on the first day, and robot-assisted gait training on the second day

The kinematic and kinetic data are shown in Table 2. We were able to measure the kinematic data of all participants. However, as it was difficult for some children to place one foot on each force plate, we analyzed the kinetic data of 7 participants for analysis.

**Table 2:** Results of maximum joint angles and ground reaction forces

		Pre	Post	p			Pre	Post	p
**RAGT**					**NAGT**				
MHF	AF	56.2 (15.2)	69.6 (16.5)	0.039^a^	MHF	AF	51.3 (15.8)	53.2 (19.5)	0.849
	UAF	61.6 (8.8)	68.5 (10.7)			UAF	58.2 (16.4)	55.1 (19.6)	
MHE	AF	−28.1 (13.7)	−35.6 (16.3)	0.144	MHE	AF	−22.1 (20.3)	−20.7 (25.6)	0.379
	UAF	−21.1 (11.3)	−25.3 (18.6)			UAF	−16.6 (24.0)	−10.7 (25.9)	
MKF	AF	67.7 (12.3)	72.8 (16.7)	0.440	MKF	AF	69.7 (10.8)	67.0 (17.3)	0.507
	UAF	67.2 (14.5)	67.5 (13.3)			UAF	68.2 (13.9)	67.4 (16.3)	
MKE	AF	−34.5 (20.2)	−37.5 (22.7)	0.634	MKE	AF	−34.1 (17.2)	−31.3 (25.6)	0.117
	UAF	−28.7 (22.0)	−28.7 (24.9)			UAF	−31.5 (22.6)	−26.1 (25.1)	
		Pre	Post	p			Pre	Post	p
**RAGT**					**NAGT**				
Fy1	AF	−45.6 (20.0)	−51.7 (23.5)	0.339	Fy1	AF	−42.4 (24.7)	− 48.8 (25.1)	0.298
	UAF	−29.8 (19.7)	−35.4 (21.0)			UAF	−35.8 (15.5)	−42.8 (29.3)	
Fy2	AF	15.5 (14.0)	23.6 (17.1)	0.122	Fy2	AF	16.9 (15.0)	20.6 (17.0)	0.356
	UAF	39.7 (16.9)	41.4 (17.3)			UAF	29.5 (12.0)	21.9 (6.8)	
Fz1	AF	351.8 (132.3)	411.1 (143.1)	0.157	Fz1	AF	377.5 (123.1)	415.1 (161.7)	0.204
	UAF	386.2 (110.9)	402.4 (153.8)			UAF	372.6 (129.5)	396.5 (140.1)	
Fz2	AF	259.8 (91.7)	275.8 (80.9)	0.367	Fz2	AF	255.8 (78.0)	271.6 (78.5)	0.896
	UAF	292.8 (89.8)	298.5 (82.4)			UAF	288.4 (88.7)	275.6 (71.5)	

Shows the value and SD of maximum joint angles and ground reaction forces. MHF and MHE of both limbs showed significant interactions between ASSIST and TIME (p < 0.05). Also, Fy2 showed a significant interaction between ASSIST and TIME (*p* < 0.05). *p*-value is for the difference between pre- and post-trials by multiple comparisons. ^a^ indicates the significant main effect between pre- and post-trials

**Fig. 2:**
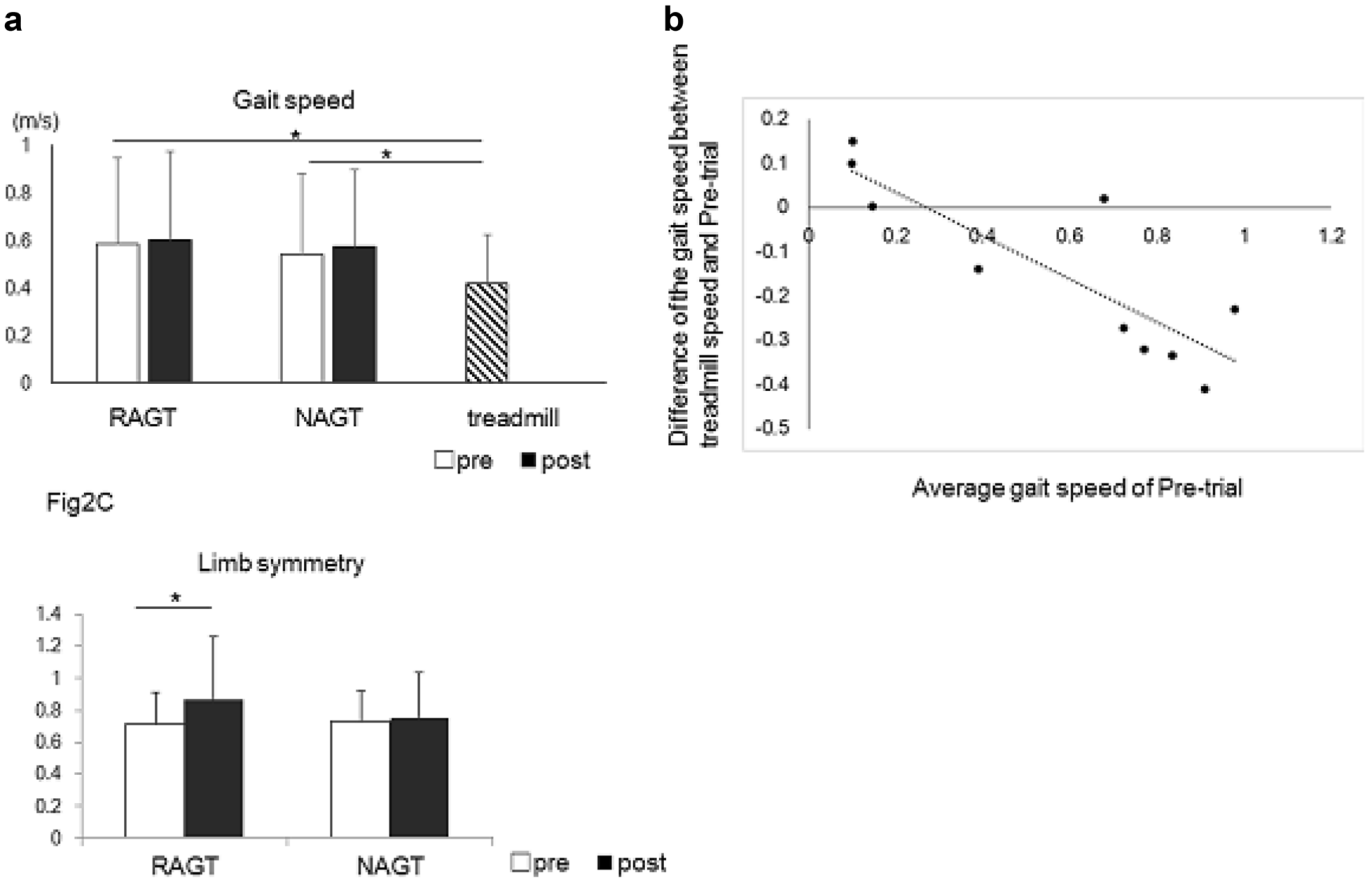
Results of gait speed and limb symmetry. **a** shows the results in gait speed. There were no significant changes in gait speed between pre- and post-trial both in RAGT and NAGT. Treadmill speed was significantly slower than the gait speed in pre-trial for both intervention days. **b** shows the correlation between the gait speed difference during treadmill and overground trials and the average difference in pre-trial gait speed. The dashed line represents the linear regression curve. **c** shows the results of limb symmetry. Limb symmetry significantly increased only after RAGT

MHF showed a significant interaction between ASSIST and TIME (F = 11.140, *p* = 0.009). In multiple comparisons, MHF did not change in the NAGT (*p* = 0.849) but significantly increased in RAGT (*p* < 0.01). MHE showed a significant main effect on SIDE (F = 8.109, *p* = 0.019) and a significant interaction between ASSIST and TIME (F = 13.408, *p* = 0.005). LS significantly increased only after RAGT (*p* < 0.05) (Fig. 2c).

The Fy2 showed a significant interaction between ASSIST and TIME (F = 12.963, *p* = 0.011). Although multiple comparisons showed no significant differences in TIME, Fy2 tended to increase in RAGT and decrease in NAGT.

The data of the gait speed is shown in Fig. 2a. The average gait speed in RAGT was 0.59 m/sin the pre-trial and 0.60 m/sin the post-trial. Also, in the NAGT, the average gait speed was 0.54 m/s in the pre-trial and 0.57 m/s in the post-trial. The average treadmill speed was 0.42 m/s and this was significantly slower than the gait speed in the pre-trial of RAGT (*p* < 0.05). Additionally, we examined the relationship between the average gait speed in pre-trial and the difference in treadmill speed and average gait speed during pre-trial. The average speed of the pre-trial of both intervention days (RAGT and NAGT) and the differences in treadmill speed and average pre-trial gait speed showed a significant negative correlation (r = − 0.85, *p* < 0.05) (Fig. 2b).

## Discussion

In this study, we compared the differences in changes of gait kinematics and kinetics in children with CP by training with and without the HWA. Our results showed significant changes in hip flexion and extension angles and limb symmetry between the RAGT and NAGT. The RAGT induced the expansion of hip joint angles on the affected side. These results are consistent with the results of previous studies that have  reported the increase of the limb symmetry after the intervention with the HWA [9]. These previous studies recruited individuals with the first stroke, who mostly have only one leg paralyzed. However, the participants recruited in this study included both diplegia and hemiplegia. The results of this study suggest that the HWA may be effective for improving the limb symmetry even in participants who have neurological problems on both limbs.

Also, these kinematic aftereffects were observed only after RAGT. This is an important phenomenon for the HWA because this device was developed to facilitate the learning effect. Usually, most studies have investigated the locomotor adaptation during gait training for more than 20 minutes [13]. In this study, the learning effect by using the HWA may be higher than other walking situations in previous studies because the effect was observed within 5 min of use. Also, other methods such as muscle strengthening [14] or Bodyweight Supported Treadmill Training [15] have failed to improve the gait pattern during walking. The results with the HWA would be useful in the methods used for improving gait kinematics in children with CP.

The Fy2 of the affected limb significantly increased after RAGT. Propulsion force has been reported to have a strong correlation with plantar flexor strength [16–18] and the leg extension angle [19]. It was believed that a decrease in propulsion force was due to the weakness of plantar flexor in healthy individuals [16] as well as in children with hemiplegic CP [20]. There was a significant increase in the Fy2 after RAGT. This change was observed intraday, supporting the possibility that the increase in propulsion force was not due to the changes in muscle strength. Instead, as leg extension angle was  reported to have a positive correlation with the propulsion force, the significant increase in hip extension angle of the affected limb is thought to be the main reason for the increase in Fy2. In contrast, during NAGT, Fy2 peak in the unaffected limb significantly decreased. Stroke individuals are reported to walk with greater demand on the non-paretic limb due to the lost function in paretic limb [21]. The children in this study might have also tried to continue walking by generating more propulsion force with the unaffected side. Therefore, the decrease in Fy2 on the unaffected limb is thought to reflect the possibility of fatigue due to over-use.

Despite the significant changes in gait kinematics and kinetics, there was no significant change in gait speed. This result is inconsistent with some previous studies which have reported a significant change in maximum walking speed of stroke individuals after 10-day intervention [9]. This inconsistency may be due to the slow treadmill speed settings because the HWA could not produce the  assistive torques to the gait with a high pitch. As shown in Fig. 2b, the average speed in pre-trial for both measurement days and the differences between treadmill settings and pre-trial gait speed showed a significant negative correlation. This result shows that we have adjusted the treadmill speed to be slower for those who walked faster on the overground. This may have enabled gait training with sufficient gait speed. As participants with higher gait speed are expected to have a better locomotor function, they might have shown more improvement in gait speed by training with faster treadmill speed.

### Limitations

This study was limited by the small sample size, which is not sufficient for generalizing the effects of the HWA for CP children. As we recruited the CP children with GMFCS I-III, this might have also affected the difference in variability of some gait parameters. Considering that gait ability differs among GMFCS levels, it would be better to adjust the number of participants for each level equally. Also, we have failed to measure the kinetic data from all participants, since placing one foot on each force plate was difficult for some children.

The treadmill speed was significantly slower than the gait speed in overground trials because of the specification of the HWA. As the HWA was primarily designed for adults, improvements in its algorithm would be needed for children’s use. Further work and follow-up studies are required to investigate the long-time effect because participants received the robot training only for about 5 min in total, which may be insufficient for locomotor learning in some participants.

## Conclusions

Children with CP walk with abnormal and asymmetry movements. In this study, we examined the changes in gait kinematics and kinetics by only assisting the hip movements with the HWA. The HWA was able to change the maximum hip angle during walking. Also, there was an improvement in the limb symmetry and the propulsion force of the affected limb, even after taking off the robot. These changes remained only after 5 min of gait training with the HWA, which supports the possibility that long-term use of the HWA may be effective for learning the adequate gait. In this study, children with CP performed RAGT on the treadmill because the treadmill has an advantage of repeating reproducible gait. Combining the HWA with the treadmill would be an effective way for gait training in children with CP.

## Data Availability

The datasets used and analyzed during the current study are available from the corresponding author on reasonable request and with permission of  Ethics Committee of Kyoto University Graduate School and Faculty of Medicine.

## Abbreviations

CP: Cerebral palsy
RAGT: Robot-assisted gait training
NAGT: Non-assisted gait training
HWA: Honda Walking Assist
MAS: Modified Ashworth Scale
ROM: Range of motion
MVC: Maximum voluntary isometric contraction
GMFCS: Gross motor function classification scale
GMFM-88: Gross motor function measure-88
PEDI: Pediatric evaluation of disability inventory
MHF: Maximum hip flexion
MKF: Maximum knee flexion
MHE: Maximum hip extension
MKE: Maximum knee extension
H-ROM: Hip-range of motion
K-ROM: Knee-range of motion
LS: Limb symmetry
